# Distribution of *Biomphalaria* Snails in Associated Vegetations and Schistosome Infection Prevalence Along the Shores of Lake Victoria in Mbita, Kenya: A Cross-Sectional Study

**DOI:** 10.24248/EAHRJ-D-19-00013

**Published:** 2019-11-29

**Authors:** Sabiano O Odero, Lilian Ogonda, David Sang, Elly O Munde, Clement Shiluli, Patrick Chweya

**Affiliations:** a Department of Biomedical Sciences and Technology, School of Public Health and Community Development, Maseno University, Maseno, Kenya; b School of Health Sciences, Kirinyaga University, Kerugoya, Kenya

## Abstract

**Background:**

Schistosomiasis due to *Schistosoma mansoni* remains a major public health problem and cause of morbidity and mortality in sub-Saharan Africa despite the implementation of control programmes. More than 6 million Kenyans are at risk of infection. Regarding control measures, *Biomphalaria* snail species, which are the obligatory intermediate hosts for transmission of *S. mansoni,* have been neglected. Mbita subcounty in Homa Bay County, western Kenya, along Lake Victoria basin, has a high prevalence of *S. mansoni* infection despite mass drug administration. This study aimed to determine the abundance of *Biomphalaria,* with their associated vegetation and schistosome infection rates, along Mbita shoreline.

**Methods:**

Sixteen purposively selected sites along the Mbita shoreline were sampled for *Biomphalaria* snails using a 30-minute scooping technique. Global positioning system technology was used to map selected sites. The associated vegetation at sampling sites were collected and identified. Schistosome infection status among the snails was determined via the detection of cercaria shedding.

**Results:**

A total of 3,135 *Biomphalaria sudanica* snails were collected. The number of snails collected differed significantly between the 16 sites (F=11.735; degrees of freedom [df]=15.836; *P*<.001). Significant mean differences (MD) were also observed in terms of the number of snails collected per vegetation type (F=7.899; df=5.846; *P*<.001). The mean number of snails collected from *Cyprus gracilis* was significantly higher than that from *Enydra fluactuants* (MD= 2.03; *P*<.001), *Eichhornia crassipes* (MD=4.15; *P*<.010), and *E. fluactuants* mixed with *E. crassipes* (MD=2.516; *P*<.010). A total of 21 (0.67%) snails shed human cercariae, while 27 (0.86%) snails shed nonhuman cercariae, despite 14 sites having human faeces contamination.

**Conclusion:**

Although the schistosome infection prevalence among the snails was low, these sites may still be important exposure sites. *C. gracilis* is the main vegetation type associated with a high abundance of *Biomphalaria* snails. Molecular techniques are necessary for verification of schistosome positivity among the snails.

## INTRODUCTION

Schistosomiasis is a parasitic disease infecting 243 million people worldwide and is endemic in 78 countries with over 85% of cases occurring in sub-Saharan Africa.^1^ About 779 million people – more than 10% of the world's population are at risk of being infected with schistosomiasis.^2^ In Africa schistosomiasis is predominantly due to *Schistosoma mansoni* and *Schistosoma haematobium* which causes intestinal and urinary schistosomiasis respectively.^3^ Schistosomiasis caused by *S. mansoni* and *S. haematobium* are the 2 species found along the Kenyan lake Victoria region,^4^ with the intermediate host being snails of the genus *Biomphalaria and Bulinus* and respectively.^5^ *S. mansoni* infection continues to be among the most important and widespread of the neglected tropical diseases in Kenya, especially among communities living around the shores of Lake Victoria in western Kenya.^4^ More than 6 million people in Kenya are infected with the disease.^6^ Previous studies among school children in Mbita and its adjacent islands of Lake Victoria have shown the prevalence to be 63.3% and 60.5%, respectively.^7,8^ Schistosomiasis has a great impact on the quality of life of individuals with important implication on the economy.^9^ *S. mansoni* infection is known to lower cognitive development in children thereby lowering their academic performance in school and reduce work force in adults.^6^ In addition, *S mansoni* infection can lead to hepatic periportal fibrosis in some patients which is a life-threatening complication.^6^

Mbita subcounty is an area on the shores of Lake Victoria whose waters are reported to be infested with intermediate snail hosts that transmit schistosomes. Most residents who live around the Lake Victoria shoreline of Mbita subcounty come into regular contact with the Lake water for their recreational activities, domestic use and are likely to be exposed to infection. Documented prevalence of *S. mansoni* remains high at approximately 60%,^10^ despite the existence of control programs against schistosomiasis including mass drug administration (MDA), education, water and sanitation programs in Mbita. Studies on schistosome infection prevalence of *Biomphalaria* snails along Lake Victoria shoreline of Mbita is necessary for identification of exposure sites in order to direct intervention to the areas.

Several species of snails of the genus *Biomphalaria* are obligatory intermediate host for the transmission of *S. mansoni*. *Biomphalaria sudanica* is the species which is commonly found along the shores of Lake Victoria while *Biompharia chaonomphala* is found in the lake bed.^9^ The natural habitat of *Biomphalaria* species except *Biomphalaria chaonomphala* are shallow stagnated water with little current near the shores of the Lakes, ponds, marshes, streams and irrigation channels.^11^ They live on water plants and mud and are most common in waters where plants are abundant and moderately polluted by organic matter such as urine and feces as is often near human habitation.^9^ Within each habitat their local distribution is usually patchy, and requires examination of different sites.^10^ Studies have shown that microhabitat factors such as protection from water flow, wave action, availability of food, presence of vegetation and stable surface for attachment influence the presence and abundance of intermediate host snails.^12^ Different species of aquatic vegetation such as water hyacinth (*Eichhornia crassipes*), water lily (*Nyamphea* spp.), hippo grass and short grasses have been associated with the presence of the intermediate snail hosts in other areas.^13^ The detection of *Biomphalaria* snails infected with schistosomes is usually performed by cercarial shedding and eventual examining of the shed cercariae under a dissecting microscope for species identification.^9^ Since *Biomphalaria* snail species act as intermediate host, knowledge on their abundance is an essential prerequisite towards the understanding *S. mansoni* disease transmission and control. However no study has been done to determine the abundance of *Biomphalaria* host snails along the Lake Victoria shoreline of Mbita, Homa Bay County. Moreover, no vegetation type has been linked with high snail abundance in this region. The purpose of the study was to determine the abundance of *Biomphalaria* with their associated vegetation and to determine schistosome infection of the snails along Mbita shoreline.

## METHODS

### Study Area

The study was conducted in Health Demographic Surveillance System (HDSS) Mbita, located in Mbita subcounty, which is 1 of the 5 subcounties of Homa Bay County in western Kenya. The area is located on the shores of Lake Victoria. The HDSS Mbita covers an area of 163.28 km^2^ and is between latitudes 0°21’ and 0°32’ South, and longitudes 34°04’ and 34°24’ East. This HDSS area comprises: Gembe East, Gembe West, Rusinga East and Rusinga West locations which are part of the 5 locations in Mbita subcounty. The HDSS in Mbita follows 11,182 households with a population of 55,929 individual.^7^ The ethnic group in this area is predominantly Luo (98%). There are 30 health facilities within the study area. The main economic activities in the area are fishing and subsistence farming. The annual rainfall in Mbita district is between 800-1900 mm. However, the rains are slightly lower in Rusinga Island with an annual range of 800 to 1,200 mm. The long rains start from March to May while the onset of short rains is from September to December. The temperatures in this region ranges from 15°C to 30°C (Mbita Strategic plan 2008-2012).

### Study Design

This was a cross sectional study that aimed at determining the abundance of *Biomphalaria* snails in 16 purposively selected study sites; assess vegetation types associated with abundance of vector snail and schistosome infection prevalence of *Biomphalaria* snails from each of the sites. Selected sites were mapped using hand held differential geographical global positioning system (GPS).

The 16 sites were purposively selected based on the following: where people had direct water contact due to their routine activities such as swimming, collecting water for domestic use, bathing, washing and occupational work like fishing or farming. The surrounding of the selected sites was assessed for the presence of human faeces.

### Collection of *Biomphalaria* Snails

Only *Biomphalaria* host snails were collected using hand held standard flat wire mesh scoop (2 mm mesh size), according to previously described method.^5,10,13^ The sampling site was measured 20m long, along the lake shore and 2m long into the main water body. The total area of the site scooped was 40 m^2^ and scooping was performed in each and every site between 7:00 am and 9:30 am. The scoop was pushed under the vegetation once, lifted up when still under the vegetation and then shaken 3 times so that the snails were dislodged from the vegetation roots onto the scoop and then the scoop was withdrawn outside. With the help of a forceps, the vector snails were picked 1-by-1 as they were counted and recorded per scoop and then put in a perforated plastic container for transportation to the laboratory. The vegetation from which each scoop was pushed under during sampling was also recorded. The total number of snails collected per site was reported in meters square (m^2^). Time of starting and ending collection per every site were noted down.

### *Biomphalaria* Species Identification

*Biomphalaria* intermediate host snails collected from the selected sites were taken to Nagasaki University Laboratory at Mbita for identification according to WHO snail identification guide.^14^

### Screening of Snails for Schistosome Infection

To determine whether the host snails were positive for schistosome cercariae, *Biomphalaria* snails were singly put in a 24 well culture plates containing 2 ml of clean and clear water and then exposed to bright artificial light for 3 hours. After the shedding period was over, the wells containing snails were put under Olympus dissecting microscope and each well with snail inside was checked for shed cercariae which had the tendency of up and down movement using forked coiled tail.^15^ Human and animal cercariae were identified based on their distinct morphological features. Nonshedding snails were returned to the aquaria until the following day when they were again exposed to bright artificial light for 3 hours and were then re-examined under the dissecting microscope before declaring them negative.

### Vegetation Associated With Distribution of *Biomphalaria* Snails

A sample of vegetation types at each sampling site was collected and taken to Kenya Marine and Fisheries Research Institute (KEMFRI), Kisumu, for identification. The vegetation types from which the snails were scooped were: Buffalo spinach (*Enydra fluactuants*), water hyacinth (*E. crassipes*) and Sedge (*C. gracilis*).

### Statistical Analysis

All statistical analyses were performed using SPSS Statistics version 23.0 (IBM Corp., Armonk, NY, USA). Analysis of variance (ANOVA) was used to compare mean differences (MDs) in snail abundance between different sites and associated vegetation type. Significant differences in mean number of snails collected per site and associated vegetation type was determined using Tukey's post hoc test.

### Ethical Considerations

The study was approved by the Maseno University Ethics review Committee (MUREC). Ethical approval reference number MSU/DRPI/MUERC/00256/15.

## RESULTS

### Abundance of *B. sudanica* Snails Per Site

Table 1 summarises the results on the numbers of snails infected with human and nonhuman cercariae as determined per site. A total of 3,135 *B. sudanica* snails were collected from the 16 different sampling sites. The number of snails collected differed significantly between the 16 sites (F=11.735; degrees of freedom [df]=15.836; *P*<.001). The lowest snail collection was realised at Bau (n=59; M=0.73) and the most at Orundu (n=356; M=11.87), with Tukey's post hoc test indicating that the mean number of snails collected at Orundu differed significantly from all other sites (*P*<.001).

**TABLE 1.** Number of *Biomphalaria* Snails and Their Schistosome Infection Prevalence

Site	Snail Count	Human Schistosomes n (%)	Nonhuman Schistosomes n (%)	Presence of Human Faeces (Positive/Negative)
Koguna	328	0	5 (1.52)	positive
W. Bur–B	238	0	4 (1.66)	positive
Orundu	356	2 (0.56)	0	positive
Kochola	177	0	3 (1.70)	positive
Ngou	187	0	2 (1.15)	positive
Wakondo	151	3 (1.50)	0	positive
Uyoga	118	4 (3.34)	2 (1.68)	positive
Kosata	174	0	3 (1.69)	negative
Kobara	139	0	0	positive
Kombe–B	339	0	0	positive
Kalea	171	0	0	positive
Nyagina	175	4 (2.30)	2 (1.15)	positive
Kombe–A	116	5 (4.67)	0	positive
Kigoda	121	0	0	positive
Bau	59	0	0	negative
W. Bur–A	286	3 (2.12)	6 (2.12)	positive
**Total**	**3,135**	**21 (0.67)**	**27** (0.86)	

Twenty-one (0.67%) of the *B. sudanica* snails shed human cercariae, and 27 (0.86%) shed nonhuman cercariae, but no significance difference was found in the proportion of snails positive for cercariae between the sites (*P*=.338).

### Schistosome Infection Prevalence of *B. sudanica* Snails Per Site

Out of the 3,135 *B. sudanica* snails that were collected, only 21 (0.67%) shed human cercariae, and 27 (0.86%) shed nonhuman cercariae. However, no significant difference was found in the proportion of snails positive for cercariae between the sites (*P*=.338). Moreover, human faeces were detected in all the sites except Bau and Kosata. The results are summarised in Table 1.

### Distribution of *B. sudanica* Snails Per Pure and Mixed Vegetation

Table 2 shows the distribution of *B. sudanica* in pure and mixed vegetation types. There were significant differences in abundance of *B. sudanica* snails based on different vegetation types. Based on pure vegetation, the mean number of *B. sudanica* snails collected from *C. gracilis* was significantly higher than those from *E. crassipes* (MD=4.15; 95%CI, 0.65 to 7.65; *P*=.01), and *E. fluactuants* (MD=2.03; 95%CI, 0.69 to 3.37; *P*<.001). There were also significant differences in the number of snails between *C. gracilis* combination with *E. fluactuants* versus *E. fluactuants* combination with *E. crassipes* (MD=4.71; 95%CI, 1.14 to 8.27; *P*=.002). These findings suggest that *C. gracilis* is the main vegetation type associated with highest number of *Biomphalaria* snails in the 16 sites studied.

## DISCUSSION

Our investigation on the vegetation types and schistosome infection of *B. sudanica* have indicated varied observations in 16 sites, in shores along Lake Victoria shores of Mbita, Kenya. The results of the current study demonstrated high abundance of *B. sudanica* host snail vectors of *S. mansoni* from the selected sites. Orundu and Bau sites had the highest and lowest snail collection respectively. Examination of distribution of snails based on vegetation type revealed that there were more snails in the *C. gracilis* relative to *E. crassipes* and *E. fluactuant* while mixed vegetation showed that *E. crassipes* together with *C. gracilis* relative to *E. fluactuants* combination with *E. crassipes* had higher number of snails.

**TABLE 2.** *Biomphalaria sudanica* Abundance According to Vegetation Type

Vegetation Type	Mean Difference	95% Confidence Interval	*P* Value
**Pure vegetation**			
CG Vs EC	4.15	0.65–7.65	.010
CG vs EF	2.03	0.69–3.37	<.001
EC vs EF	−2.17	−5.50–1.27	.474
**Mixed vegetation**			
ECCG vs EFCG	−1.91	−5.70–1.96	.722
ECCG vs EFEC	2.80	−1.63–7.23	.464
EFCG vs EFEC	4.71	1.14–8.27	.002

Mean differences in the distributions of *Biomphalaria* snails according to pure vegetation and mixed vegetation were determined using Tukey's post hoc test. Abbreviations: CG, *Cyprus gracilis*; EC, *Eichhornia crassipes*; EF, *Enydra fluactuants*; ECCG, *Eichhornia crassipes*/*Cyprus gracilis*; EFCG, *Enydra fluactuants*/*Cyprus gracilis*; EFEC, *Enydra fluactuants*/*Eichhornia crassipes*

This study results concur with that of Standley and coworkers^16^ on the distribution of *Biomphalaria* (Gastropoda: Planorbidae) along the shoreline of Lake Victoria of Kenya, Uganda and Tanzania that showed not only are *B. sudanica* snails widely found around the shoreline of Lake Victoria, but also their distribution is heterogeneous on a local scale. This however contradicts a previous study,^10^ which concluded that there could be only about 10-15 snails per site sampled. Furthermore, this study showed that the number of snails collected after 30 minutes of scooping in an area of 40 m^2^ from the selected sites was more compared to other studies. The lower numbers could be due to improper snail sampling techniques which is not standardised, lack of proper identification of vegetation preferred by snails or the area of the site covered. It has earlier on been demonstrated that snails have preference to vegetation from where they get shelter, protection from the waves and also egg laying.^16^ From this study, it is scientifically plausible that since *B. sudanica* vector snails are found in the vegetation along the shoreline next to the open beaches where people frequent for fishing, water harvesting and swimming, such places may be important transmission points taking into account that human faeces were also found around the selected sites.

In the current study, we further demonstrated that of the 3,135 snails collected only 0.67% shed human cercariae while 0.86% shed nonhuman cercariae. The result shows that there was very low percentage of *B. sudanica* host snails shedding human cercariae even though all the sites except 2 had human faeces around them. It would be important that future study includes collection and *S. mansoni* eggs examination of faeces from site residents to help confirm possibility of *S. mansoni* foci of transmission. Taking into account that our current study area has high schistosomiasis transmission,^8^ it was rather surprising to find that very few vector snails shed human cercariae. It is important to appreciate that presence of the snail does not necessarily mean that they are also infected. However, the findings of this study is consistent with other previous study results from endemic areas with high transmissions where snail vector infection with cercariae were low and even some places had no single snail shedding cercariae^17^ on host snail vectors of *S. mansoni*. This study further concurs with earlier study findings^18^ which revealed that in the Lake Victoria basin Western Kenya, only 1.04% of the total collected vector snails from different sites shed cercariae. Furthermore, an additional epidemiological study in Uganda Sesse island, did not find any *Biomphalaria* snails shedding schistosome cercariae.^19^ Studies continue to come up with explanation concerning these findings: First, the development of parasite inside the vector snails has stages.^20^ After penetration of the miracidium into the host snail, it develops into primary and secondary sporocysts that hence liberate mature cercariae and this takes approximately 4 weeks,^15^ so it depends at what stage the vector snail was collected. Secondly, in the prepatent period very few snails will shed cercariae,^21^ therefore identification of vector infection cannot be performed by classical detection (shedding).^9^ Although classical detection is routinely used in the laboratories for identification of human schistosome-infected vector snails, few snails are always found shedding human cercariae and it is worth noting that molecular techniques could be the most accurate method in determining positive *Biomphalaria* host snails both in their prepatent and patent stages of infection.^22–24^

In determining the abundance of snails based on the vegetation types, the results revealed that there were higher number of snails in the *C. gracilis* relative to *E. crassipes* and *E.fluactuants*. This finding implies snails’ preference to *C. gracilis.* When mixed vegetation was compared, *E. crassipes* together with *C. gracilis* relative to *E. fluactuants* combination with *E. crassipes* had higher number of snails. In addition, a study by Angelo and colleagues^25^ showed that the abundance and disease transmission potential of snail intermediate host of human schistosomiasis in fishing community of Mwanza were common in *E. crassipes.* On the contrary, more snails have been shown to be found in *Echinochloa stagnina* mixed with *E. crassipes* along the shoreline of Lake Victoria.^5^ Rather than feeding directly on higher plants, snails are known to feed by grinding the decaying plants matter, microflora, algae and bacteria that covers the vegetation.^26^ Therefore *C. gracilis* being soft grass, the rate of drying and decaying is high and the growth of algae and other microflora on the decaying materials will be on the increase. Due to the availability of food -especially algae, the population of vector snails is most likely to rise. We therefore, from our current study demonstrate that the *C. gracilis* is the vegetation of choice for the vector snails. Moreover, its combination with *E. fluactuants* could be more favourable for snails. Further studies should consider vegetation as important determinants snail distribution.

## CONCLUSION

The study revealed that *C. gracilis* is the main vegetation type associated with a high abundance of *Biomphalaria* snails. Although schistosome infection prevalence among the snails was low, these sites may still be important exposure sites. Molecular techniques are necessary for the verification of schistosome positivity among snails.
